# Formulation Optimization of Erythromycin Solid Lipid Nanocarrier Using Response Surface Methodology

**DOI:** 10.1155/2014/689391

**Published:** 2014-06-18

**Authors:** Anil Kumar Sahu, Tekeshwar Kumar, Vishal Jain

**Affiliations:** University Institute of Pharmacy, Pt. Ravishankar Shukla University, Raipur 492010, India

## Abstract

In present work response surface methodology (RSM) using the miscellaneous design model was used to optimize formulations of erythromycin solid lipid nanocarriers (ERY-SLN). Two-factor three level factorial design was considered for optimization. There were three parameters, drug entrapment efficiency (EE), drug loading (DL) percentage, and mean particle size of ERY-SLN, considered for investigating the optimal formulation with respect to two independent variables, including lipid concentration (*X*_1_) and surfactant : cosurfactant ratio (*X*_2_). The result showed that the optimal ERY-SLN was composed of lipid concentration (*X*_1_) 15 mg/mL and surfactant : cosurfactant ratio (*X*_2_) 1 : 1 with %EE of 88.40 ± 2.09%, DL of 29.46 ± 0.69%, mean particle size of 153.21 ± 2.31 nm, polydispersity index (PDI) of 0.026 ± 0.008, and zeta potential value of −15.18 ± (−5.53)  mV. DSC and TEM study showed that there was no chemical interaction between ERY and lipid (GMS) and the ERY-SLN particles are nonspherical, respectively. The drug release experiments exhibited a sustained release over during 24 h, up to 66.26 ± 2.83%. Accelerated stability studies showed that there was no significant change occurring in the responses after storage condition for a total period of 3 months.

## 1. Introduction

Erythromycin 9-{O-[(2-methoxyethoxy)methyl]oxime} (ERY) is a 14-member lactone ring macrolide antibiotic produced from a strain of the actinomycete* Saccharopolyspora erythraea*, formerly known as* Streptomyces erythraeus* [1]. It has been used clinically for over 60 years. It is active in a wide variety of infections like bronchitis, severe campylobacter enteritis, chancroid, diphtheria, legionnaires, pneumonia sinusitis, trench fever, chlamydia, syphilis, acne, and gonorrhea [2]. ERY has poor solubility in water, instability in gastric pH, unpleasant taste, low half-life (1–1.5 hrs), and low oral bioavailability (about 35%). Due to the above reasons its oral application is limited [3]. ERY topical preparations are used as a second-line topical treatment for acne following failure of nonantibiotic topical preparations to treat the condition. Sometimes resistance to ERY may be developed by many bacterial strains due to different mechanisms, among which an impaired permeability of the bacterial cells, resulting in a reduction of the drug concentration in the cytoplasm to insufficient (subactive) levels. Topical administration of drug might be beneficial for the treatment of skin diseases because it reduced the systemic side effects and improves the patient compliance, but topical administration of drug is still a challenge in drug delivery due to the difficulties in controlling the fate of drug within the skin [4]. For overcoming the above difficulties lipid nanoparticles have shown a great potential as a carrier for topical administration of active pharmaceutical ingredients.

Solid lipid nanoparticles (SLN) are the forerunner of the rapidly developing field in nanotechnology with several potential applications in drug delivery, clinical medicine, and research [5]. The SLNs offered a great potential for the administration of active molecules by any route of administration and simultaneously having other advantages like improved avoidance of organic solvent's biotoxicity, drug stability, high drug payload, and incorporation of lipophilic and hydrophilic drugs. SLN can be also used to improve the bioavailability and to obtain sustain release of drugs [6]. SLN administered through topical route has advantages such as achieving the higher amount of drug concentration in subjected area and minimizing the systemic transport of drug, which bypasses first-pass metabolism and systemic toxicity.

Experimental design and optimization of pharmaceutical formulation are a key issue for the development of nanocarriers. Optimization procedure may help to develop nanocarriers having maximum drug entrapment efficiency and drug loading capacity and appropriate mean particle size through minimum experimental trials. Presently computerized optimization technique based on response surface methodology (RSM) is used for optimization purpose. Response surface methodology is a collection of mathematical and statistical techniques based on polynomial equation, which must describe the influence of independent variables on response with the objective of making statistical previsions [7].

The objective of this study was to use response surface methodology (RSM) in conjunction with miscellaneous design to establish the functional relationship between operating variables and responses. Glyceryl monostearate, Poloxamer 188, and soya lecithin were selected as the solid lipid, surfactant, and cosurfactant, respectively. ERY-SLN was prepared using hot homogenization method followed by ultrasonication. Optimization was done by computer simulation programme Design-Expert version 8.0.1. SLN was further characterized for their mean particle size, loading parameters,* in vitro* drug release behaviour, and morphology.

## 2. Experimental

### 2.1. Materials

Erythromycin (ERY) and Poloxamer 188 were a generous gift from S Kant Health Care Ltd., Gujarat and Signet Chemical Corporation Pvt. Ltd., Mumbai, India, respectively. Glycerol monostearate (GMS), cetyl alcohol (CA), cetostearyl alcohol (CSA), and stearic acid (SA) were purchased from Loba Chemie Pvt. Ltd., Mumbai, India. Soya lecithin was purchased from HiMedia Lab. Pvt. Ltd., Mumbai, India. All other chemicals and solvents used for the study were of analytical grade.

### 2.2. Partitioning Behaviour of ERY in Different Lipids

ERY (25 mg) was dispersed in a mixture of melted lipid (2 g) and hot distilled water (2 mL). The mixture was shaken for 30 min at 80°C in a hot water bath and then centrifuged at 5000 rpm for 10 min. Aqueous phase was filtered through membrane filter with a pore size of 0.45 *μ*m. The drug concentration in the water was determined by UV-spectroscopy. Partition coefficient value is determined to study its partitioning behaviour with different lipids [8].

### 2.3. Preparation of ERY Loaded SLNs

Drug-SLNs were prepared using hot homogenization followed by ultrasonication method. GMS was considered as a lipid phase; its concentration at different levels was shown in Table 1. ERY (100 mg) was dissolved in melted lipid phase, and this solution was then dissolved in 20 mL of ethanol : acetone (1 : 1) mixture. Surfactant (Poloxamer 188) and cosurfactant (soya lecithin) were dissolved in 20 mL of distilled water to obtain 2% solutions and heated up to 80°C in a beaker. When a clear homogenous lipid phase was obtained, the hot aqueous surfactant, cosurfactant solution were added to hot lipid phase, and homogenization was carried out at 15,000 rpm, for 10 min, using a high-speed homogenizer (T10 basic, IKA-Werke GmbH & Co. KG, Staufen, Germany) with maintained temperature at 80°C The resulted preemulsion was ultrasonified using a probe sonicator (Frontline Sonicator) at 50 W for 5 min. Later, the mixture was cooled to room temperature and diluted up to 100 mL with deionised water yielding ERY-SLNs dispersion [9].

### 2.4. Determination of Optimal Concentration of Surfactant

ERY (50 mg) was dissolved in 100 mg melted lipid (GMS) and this solution was then dissolved in 20 mL of ethanol : acetone mixture (1 : 1). Surfactant was added to cosurfactant aqueous dispersion at concentration of 1%, 2%, 3%, 4%, and 5% (w/v). SLNs were prepared as in the above-mentioned method. The most favourable concentration of surfactant was determined from size, entrapment efficiency (EE), and drug loading (DL) of the prepared SLNs [10].

### 2.5. Experimental Design

Statistical models are extensively used to design the formulation of lipid based nanoparticles; it was essential to recognise the independent variables in the formulation which can affect the properties of desired formulation. A 3-level factorial-response surface methodology (3LF-RSM) was used to study the effect of different variables on formulation properties like mean particle size, percentage drug loading (%DL), and entrapment efficiency (%EE) of the prepared SLNs. Independent variables include lipid concentration (*X*_1_) and ratio of surfactant: cosurfactant (*X*_2_) (Table 1). The best fitted model for statistical analysis was considered significant when *P* value <0.05. Predicted *R* ^2^ value and ANOVA were pursued to confirm best fittingness of the model. Three-dimensional (3D) surface plots were used to establish the relationship between independent variables and dependent variables (response). The desirability function of particle size was in the minimum level while that of entrapment efficiency and drug loading was in the maximum level, which was used for optimization of formulations [7].

### 2.6. Determination of Mean Particle Size, PDI, and Zeta-Potential of the ERY-SLNs

The mean particle size (*z*-average) of the SLNs and polydispersity index (PDI) as a measure of the width of particle size distribution is found out by photon correlation spectroscopy (PCS) using a Zetasizer (Nano ZS 90, Malvern Instruments, UK) at 25°C and a 90° scattering angle. SLNs formulation was diluted with double distilled water to weaken opalescence before measurements. The surface charge was assessed by measuring the zeta potential of SLNs based on the Smoluchowski equation, using the same equipment at 25°C with electric field strength of 23 V/cm [11].

### 2.7. Determination of %EE and %DL of the ERY-SLNs

Percentage of EE and DL was evaluated by determining the amount of free ERY in the aqueous surfactant-cosurfactant solution which was separated by using the cooling centrifuge (Remi Instruments Ltd., Mumbai, India) [12]. The ERY-SLNs aqueous dispersion was placed in the cooling centrifuge tubes and speed of centrifuge was kept at 12,000 rpm for 20 min at 4°C. The concentration of ERY in the aqueous phase was determined using UV-visible spectrophotometer (UV 1700, Shimadzu, Japan) at *λ* max 236 nm. The %EE and %DL were calculated by the following equations:

(1) %EE =Weight  of  ERY  used−Weight  of  free  ERYWeight  of  ERY  used×100,%DL =Weight  of  ERY  used−Weight  of  free  ERYWeight  of  GMS×100.

### 2.8. Differential Scanning Calorimetric (DSC) Study

DSC analysis was carried out by using DSCQ20 (TA Instruments, USA) at a heating rate of 10°C/min in the range of 40–220°C. DSC studies were conducted for ERY, GMS, and physical mixture of ERY and GMS in ratio 1 : 1 and freeze-dried ERY-SLNs of the optimized batch [13].

### 2.9. Transmission Electron Microscopy (TEM) Study

The surface morphology and size of optimized SLNs were analyzed by transmission electron microscopy (TEM). It optimized ERY-SLNs aqueous dispersion which was placed on copper grids coated with carbon film and dried at room temperature for observation. The magnification for the TEM images was 150000x [14].

### 2.10. Determination of* In Vitro* Drug Release from Optimized ERY-SLNs

*In vitro* release of ERY from optimized ERY-SLNs was determined by modified Franz's diffusion using dialysis membrane (molecular weight cutoff 10,000 Da). Dialysis membrane was kept in double distilled water for 24 hours before utilizing in modified Franz's diffusion cell. ERY-SLNs aqueous dispersion (2 mL) was placed in the donor compartment, and the receptor compartment was filled with dissolution medium (pH 6.8 phosphate buffer) and maintained temperature at 32 ± 0.5°C by continuous stirring at 100 rpm. After regular time intervals, samples were withdrawn from the receptor compartment and exact volume of a dissolution medium was added to the same compartment to maintain the constant volume throughout the study. The amount of ERY released was analyzed by UV-visible spectroscopy [15].

### 2.11. Storage Stability Studies

The storage stability studies were carried out with the optimized ERY-SLN formulation. A 10 mL of ERY-SLN dispersion with 2 mg/mL drug concentration was taken into glass vials and stored at 4 and 25°C for 3 months. The stability test was analyzed on the basis of particle size, zeta potential, and percentage entrapment efficiency determination in the dispersion with a sampling frequency of 1 month.

### 2.12. Statistical Analysis

Design-Expert software (version 8.0.1; Stat-Ease, trial version) was utilised for statistical analysis and graph plotting. The results of one-way analysis of variance (ANOVA) for the dependent variables were utilised for the selection of the model which could be considered significant for the response variables.

## 3. Results and Discussions

### 3.1. Partitioning Behaviour of ERY

Standard curve of ERY in methanol was utilised for estimating the concentration of the ERY in the aqueous phase. Partition coefficients obtained were 110.96 ± 27.90, 49.77 ± 2.10, 19.18 ± 0.77, and 45.79 ± 4.65 for glycerol monostearate, stearic acid, cetyl alcohol, and cetostearyl alcohol, respectively. On the basis of results, it can be claimed that the lipophilic drug ERY is soluble in the greater amount in GMS than any other lipid used. Thus, the GMS used as the lipid phase for the study of ERY-SLNs.

### 3.2. Preparation of ERY-SLNs

The high shear homogenisation followed by ultrasonication method is the easy method which can be utilised for the laboratories' production of the SLNs. 20 mL ethanol : acetone (1 : 1) was incorporated for homogenous distribution of ERY inside the lipid phase (GMS). The homogenisation speed and sonication time were optimized to 15,000 rpm for 10 minutes and 5 minutes at 50 W, respectively.

### 3.3. Determination of Optimal Concentration of Surfactant

The surfactant concentration plays a significant role in desired formulation properties. On the basis of preliminary studies, the surfactant concentration from 1% to 5% (w/v) Poloxamer 188 was selected for evaluating the effect of surfactant concentration on mean particle size, percentage of drugs EE and DL. Consequently, the smallest particle size, highest %EE and %DL were found to be 2% (w/v) Poloxamer 188. At this concentration, the mean particle size, %EE, and %DL of SLNs were 138.42 ± 17.48, 84.52 ± 5.58, and 28.17 ± 1.86, respectively (Table 2). It is concluded from the above results that the 2% Poloxamer 188 was determined as optimal concentration.

### 3.4. Optimization Data Analysis for the ERY-SLNs

Observed responses of nine formulations were fitted to various models by using Design-Expert software trial version 8.0.1. It was seen that the quadratic models were best-fitted for the studied responses, that is, mean particle size, %EE, and %DL. The quadratic equations generated for responses were given as

(2) Mean  particle  size (nm)=−672.35+70.06X1+15−24X2  +0.64X1X2−1.04X12+70.50X22,Entrapment  Efficiency (%)=+85.68+0.16X1−4.08X2  +0.13X1X2+0.01X12−6.66X22,Drug  Loading (%) =+66.23−3.26X1−1.66X2    +0.06X1X2+0.05X12−1.79X22,

 where *X*_1_ and *X*_2_ represent the coded values of the lipid concentration and surfactant cosurfactant ratio, respectively. The positive value of a factor in the above equations point outs the enhancement of that response and vice versa. All values of correlation coefficient (*R* ^2^), SD, % coefficient of variation, and results of ANOVA are shown in Table 3. A value of *R* ^2^ and results of ANOVA for the dependent variables confirmed that the model was significant for observed response variables.

Predicted optimum ranges of the independent variables were listed in Table 4. The fitting results point out that the optimized SLNs formulation with high EE, high drug loading percentage, and small mean diameter was obtained at the lipid concentration of 15 mg/mL and ratio of surfactant: cosurfactant of 1.07 : 1, respectively. Table 4 showed that the observed values of the prepared batch with the optimized formula was very close to the predicted values, with low percentage bias, suggesting that the optimized formulation was trustworthy and rational.

### 3.5. Response Surface Plots

**Figure 1:**
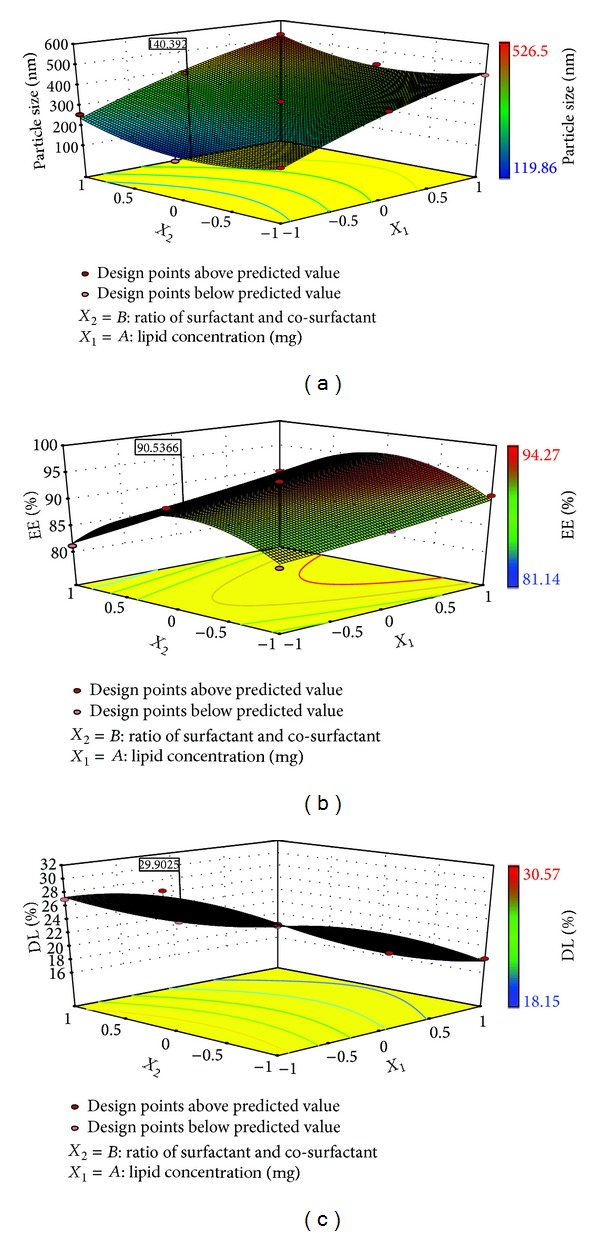
Response surface plot showing the effect of lipid (GMS) concentration (*X*_1_) and ratio of surfactant: cosurfactant (*X*_2_) on (a) mean diameter of particles (*Y*_1_), (b) %EE (*Y*_2_), and (c) %DL (*Y*_3_).

The relationship between the dependent and independent variables is further elucidated by constructing the response surface plot. The three-dimensional (3D) response surface graphs generated by the Design-Expert software (trial versions version 8.0.1) for the most statistical significant variables on the evaluated parameters are presented in Figure 1. The three-dimensional (3D) response surface curves are used for studying the interaction patterns. On the basis of three-dimensional response surface graphs, it can be said that the lipid concentration and surfactant: cosurfactant concentration produces a significant effect on mean particle size, EE and DL percentage. Graphs show that with increasing the concentration of lipid in formulation, mean particle size and EE% increase but DL% was decreased and vice versa. In case of second factor (surfactant-cosurfactant ratio) optimum concentration, ratio (1 : 1) was responsible for minimum mean particle size and higher EE and DL%. The response surface graphs represented that the taken dependent variables (lipid concentration and surfactant: cosurfactant ratios) were statically significant.

### 3.6. Particle Size, PDI, and Zeta Potential of ERY-SLNs

Particle size, PDI, and zeta potential of the ERY loaded SLNs are depicted in Table 3. The mean particle sizes, PDI values, and zeta potential of the total nine formulations were obtained to be in the range of 119–526 nm, 0.024–0.458, and −7.54 to −19.57 mV, respectively.

#### 3.6.1. Effect of Surfactant Cosurfactant Ratio on the Particle Size, PDI, and Zeta Potential

Different ratio of surfactant and cosurfactant combinations produced the very predominant effects on mean particle size and stability of the ERY-SLNs dispersion. A higher surfactant and cosurfactant concentration reduces the surface tension, prevents the particle agglomeration, and decreases the mean particle size. Results showed that the mean particle size was to decrease with an increase the surfactant concentration up to the optimal ratio (1 : 1) in combination mixture with cosurfactant; after that further increase in the surfactant concentration produced inverse effects, that is, increase in the mean particle size at a constant amount of lipid and the case of cosurfactant concentration vice versa. Thus, this implies that the increased surfactant and cosurfactant concentration at the optimal level the mean particle size is significantly reduced. On the basis of observed PDI values, it can be concluded that the surfactant and cosurfactant concentration did not produce any considerable effect on PDI values of different studied formulations. Zeta potential is the measure of overall charges got hold on by particles in a particular medium and is allowed to predict about the stability of colloidal dispersion through electrostatic repulsion between the charged particles. Observed zeta potential values indicate that the surface charge of the particles in different formulation was negative. There was no significant correlation between the zeta potential and surfactant: cosurfactant ratio.

#### 3.6.2. Effect of Lipid Concentration on the Particle Size, PDI, and Zeta Potential

The concentration of lipid has the significant effect on the particle size of the SLNs formulation because it solubilises the drug in formulation. Response surface plots (Figure 1(a)) show that, with the increase in GMS (lipid) concentration from 15 mg/mL to 25 mg/mL, the mean particle size of the formulation was also increased in each case (i.e., directly proportional). Formulations SLN5, SLN6, SLN7, and SLN8, which contained the highest amount of lipid, comparatively showed the larger particle size (in the range of 380–550 nm) than that of formulations containing the low amount of lipid (in the range of 100–350 nm). The zeta potential of formulations also changes with increasing the amount of lipid (GMS) from −19.57 mV to −7.54 mV.

### 3.7. Percentage Entrapment Efficiency (EE) and Drug Loading (DL) of ERY-SLNs

Percentages of entrapment efficiency (EE) and drug loading (DL) of the ERY loaded SLNs are depicted in Table 3. The entrapment efficiency (EE) and drug loading (DL) percentages of the total nine formulations were obtained to be in the range of 81.14%–94.27% and 18.15%–30.57%, respectively. Nature of the drug plays a significant role in the EE and DL capacity because the drug is encapsulated in lipidic phase. ERY is a lipophilic drug, and its solubility is also higher in GMS (conclusion drawn from partition coefficient study), so that the %EE noticeably was found to be higher.

#### 3.7.1. Effect of Surfactant-Cosurfactant Ratio on Percentage of EE and DL

The surfactant and cosurfactant concentration produced a remarkable effect on the EE and DL% (Figures 1(b) and 1(c)). Surfactant and cosurfactant ratio plays a major role in the solubility of drug in external phase (i.e., aqueous phase). Optimum surfactant and cosurfactant concentration ratio (1 : 1) shows the high EE and DL% in reference to other two ratios (1 : 2, 2 : 1) (Table 1).

#### 3.7.2. Effect of Lipid Concentration on Percentage of EE and DL

The lipid concentration produced a significant effect on the %EE. Response surface curve showed that increased the lipid concentration in formulation showed higher %EE. This may be due to the increasing internal phase; more amounts of lipid were available for the dissolving of drug (ERY). ERY has the highest partition coefficient in GMS (conclusion drawn from partition coefficient study) which is another reason for increasing %EE.

### 3.8. DSC Studies

**Figure 2:**
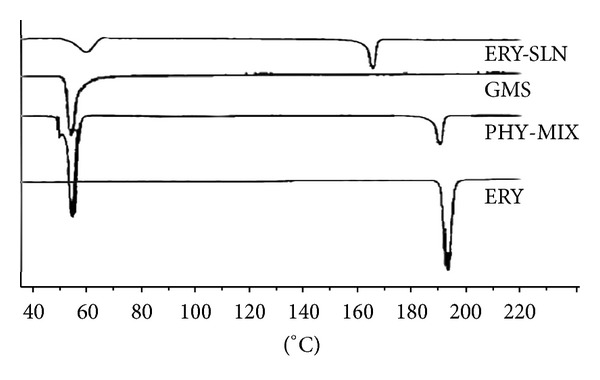
DSC thermograms of pure Erythromycin (ERY), physical mixture of ERY and GMS (PHY-MIX), bulk GMS (GMS), and ERY loaded lyophilised SLNs (ERY-SLN).

DSC studies were performed for the assessment of the drug excipients interactions. The DSC thermogram was run for the pure ERY, bulk GMS, physical mixture of ERY and GMS at 1 : 1 ratio, and drug loaded freeze dried SLNs.  Figure 2 showed the DSC thermogram. The melting point of ERY is 193–195°C. The DSC thermogram of ERY and bulk GMS showed a sharp endothermic peak at 193°C and 55°C, respectively. No significant shift in the position of endothermic peaks was observed after running the physical mixture (1 : 1) of ERY and GMS. Thus, no chemical interaction was found between ERY and GMS. ERY loaded SLNs show two endothermic peaks in DSC thermogram; first one was observed at 56°C for GMS and second one at around 165°C for mannitol (cryoprotectant), but ERY peak was not found. This result suggests that ERY entrap in SLNs exists in the amorphous state.

### 3.9. Transmission Electron Microscopy (TEM) Studies

**Figure 3:**
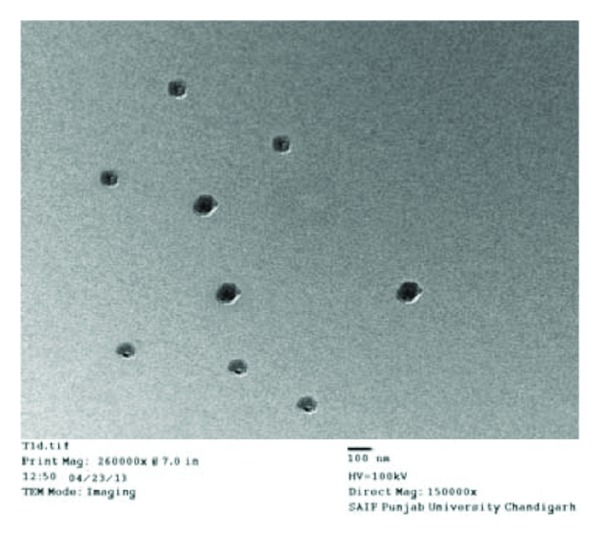
Transmission electron microscopic (TEM) image of optimized Erythromycin loaded solid lipid nanoparticles.

In order to investigate the morphology and size of the optimal ERY-SLNs, TEM was used. TEM photomicrograph of the ERY loaded SLNs is shown in Figure 3. The optimized ERY-SLNs formulation showed nonspherical shape and particle size is about 150 nm which are almost the same results obtain from Zetasizer determination.

### 3.10. *In Vitro* Drug Release Studies

**Figure 4:**
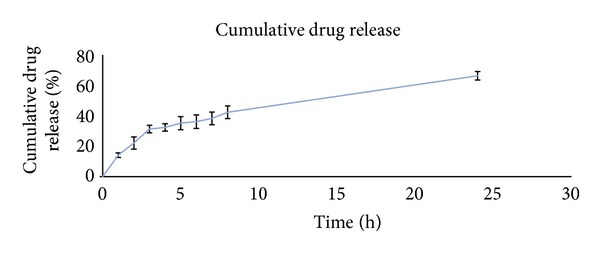
Release curve of the optimized ERY-SLNs suspension in 6.8 pH phosphate buffer at 37°C.

The* in vitro* release curve of the optimal ERY-SLNs suspension in 6.8 pH phosphate buffer at 32 ± 0.5°C was shown in Figure 4. Cumulative percentage drug release of optimized ERY-SLNs suspension showed 66.26 ± 2.83% in 24 hours.* In vitro* release curve showed the initial burst release with the about 40% of drug release during the first two hours; after that release sustained from the optimized ERY-SLNs. Burst release occurred due to the presence of the free ERY in the external phase and on the surface of the SLNs. The lipophilic nature of the ERY could be the reason for sustained release of the drug from internal lipidic phase after initial burst release. Initial burst release rate was affected by the change of concentration of lipid and surfactant in external phase. When the lipid concentration increased, the initial burst release rate decreased; this may be due to the higher concentration of drug presence in the inner core. Whereas surfactant concentration increases, the initial burst release rate increases due to the increased solubility of drug in external phase.

### 3.11. Storage Stability Studies

Storage stability studies were conducted on optimized SLNs using the particle size, zeta potential, and EE as the prime parameters. There was a negligible or slight increase in the particle size during the three-month storage at 4°C and 25°C from the 153.21 ± 2.31 nm to 151.98 ± 1.68 nm and 158.81 ± 3.28 nm, respectively. In case of zeta potential similar results were seen for three months storage at 4°C and 25°C from the −15.18 ± (−5.53) mV to −14.10 ± (−1.76) mV and −12.53 ± (−0.59) mV, respectively. The EE % of the optimized batch initially was found to be 88.40 ± 2.09% while that after three-month storage at 4°C and 25°C was found to be 87.06 ± 0.53% and 85.69 ± 0.51%, respectively, indicating that the drug can retain within the SLNs for the sufficient period of time. On storage of the SLNs, there was no significant change occurring in the size, zeta potential, and EE% of the SLNs. Hence, they were found to be stable on taken storage condition (at 4°C and 25°C) for a total period of 3 months.

## 4. Conclusion

The ERY-SLNs were optimized using the miscellaneous design-response surface methodology by fitting a second-order model to the response data. The effect of two variables, including lipid concentration (*X*_1_) and ratio of surfactant : cosurfactant (*X*_2_) with their interactions, had been evaluated and modelled. The best local maximum of entrapment efficiency (88.40 ± 2.09%) and minimum particle size (153.21 ± 2.31 nm) were found at lipid concentration 15 mg/ml and surfactant/cosurfactant ratio 1.07 : 1. The release profile of the produced SLN was investigated in phosphate buffer media, and it showed prolonged release during 24 h, up to 66.26 ± 2.83% release. The drug release behaviour from the SLNs exhibited a biphasic pattern with the burst release at the initial stage and sustained release subsequently. These results indicated that the SLNs obtained in this study could potentially be exploited as a carrier with an initial dose and prolonged release when therapeutically desired.

## Figures and Tables

**Table 1:** Independent variables along with their coded level, actual level, and respective responses values of different batches of ERY-SLNs.

Form. code	Coded level		Actual level		Responses				
	*X*_1_	*X*_2_	*X*_1_	*X*_2_	Mean particle size (nm)	Polydispersity index (PDI)	Zeta Potential (mV)	EE (%)	DL (%)
SLN 1	−1	−1	15	01 : 02	193.16 ± 18.49	0.024 ± 0.002	−7.54 ± (−1.41)	84.96 ± 4.17	28.32 ± 1.39
SLN 2	−1	0	15	01 : 01	119.86 ± 6.54	0.075 ± 0.030	−15.18 ± (−5.53)	91.72 ± 0.98	30.57 ± 0.32
SLN 3	−1	1	15	02 : 01	255.73 ± 22.68	0.042 ± 0.028	−11.11 ± (−3.20)	81.14 ± 1.94	27.04 ± 0.64
SLN 4	0	−1	20	01 : 02	358.20 ± 27.00	0.352 ± 0.059	−11.61 ± (−1.46)	87.60 ± 0.93	21.89 ± 0.23
SLN 5	0	0	20	01 : 01	321.46 ± 17.99	0.195 ± 0.057	−8.87 ± (−3.78)	93.39 ± 3.90	23.34 ± 0.97
SLN 6	0	1	20	02 : 01	389.20 ± 33.34	0.419 ± 0.090	−16.26 ± (−1.89)	84.55 ± 1.57	21.13 ± 0.39
SLN 7	1	−1	25	01 : 02	451.00 ± 38.00	0.286 ± 0.056	−14.56 ± (−6.03)	90.78 ± 1.46	18.15 ± 0.29
SLN 8	1	0	25	01 : 01	434.06 ± 21.43	0.208 ± 0.079	−10.23 ± (−6.77)	94.27 ± 2.98	18.85 ± 0.60
SLN 9	1	1	25	02 : 01	526.50 ± 25.12	0.458 ± 0.124	−19.57 ± (−4.76)	89.72 ± 2.81	18.20 ± 0.19

**Table 2:** The effect of surfactant (Poloxamer 188) concentration (%w/v) on mean particle size, %EE, and %DL of ERY-SLNs.

Lipid	Poloxamer 188 concentration (%w/v)	Mean particle size (nm)	EE (%)	DL (%)
GMS	1	337.96 ± 9.81	79.99 ± 4.89	25.99 ± 1.63
	2	138.42 ± 17.48	84.52 ± 5.58	28.17 ± 1.86
	3	224.29 ± 11.69	79.14 ± 3.69	26.37 ± 1.23
	4	338.76 ± 9.51	68.42 ± 5.06	22.80 ± 1.68
	5	387.29 ± 9.15	67.47 ± 5.60	22.48 ± 1.86

**Table 3:** Summary of results of regression analysis for responses and analysis of variance for particle size, EE, and DL.

Parameters	DF	SS	MS	*F*	*P* value	*R* ^2^	SD	Coeff. of variance %
Mean particle size								
Model	5	1.35*E* + 05	26902.5	49.04	0.0045 **significant**	0.9879	23.42	6.91
Residual	3	1645.67	548.56					
Total	8	1.36*E* + 05						

%Entrapment efficiency								
Model	5	149.33	29.87	11.56	0.0356 **significant**	0.9507	1.61	1.81
Residual	3	7.75	2.58					
Total	8	157.08						

%Drug loading								
Model	5	168.89	33.78	61.66	0.0032 **significant**	0.9904	0.74	3.21
Residual	3	1.64	0.55					
Total	8	170.53						

**Table 4:** Comparison of the observed and predicted values in the SLN prepared under predicted optimum conditions.

S. number	Response variables	Predicted optimum range		Predicted value	Observed value	Bias %
		*X*_1_ (mg/mL)	*X*_2_			
1	Mean particle size (nm)	15	1.07 : 1	144.59	153.21	−5.96
2	%EE	15	1.07 : 1	90.21	88.40	2.00
3	%DL	15	1.07 : 1	29.78	29.46	1.07
4	PDI	15	1.07 : 1	—	0.026	—
5	ZP	15	1.07 : 1	—	−15.18	—
